# Exploring the fear of clinical errors: associations with socio-demographic, professional, burnout, and mental health factors in healthcare workers – A nationwide cross-sectional study

**DOI:** 10.3389/fpubh.2024.1423905

**Published:** 2024-06-26

**Authors:** Laurent Boyer, Albert W. Wu, Sara Fernandes, Bach Tran, Yann Brousse, Tham Thi Nguyen, Dong Keon Yon, Pascal Auquier, Guillaume Lucas, Bastien Boussat, Guillaume Fond

**Affiliations:** ^1^CEReSS—Health Service Research and Quality of Life Center, UR3279, Aix-Marseille University, Marseille, France; ^2^Department of Public Health, Assistance Publique-Hôpitaux de Marseille, Marseille, France; ^3^Johns Hopkins University Bloomberg School of Public Health, Johns Hopkins School of Medicine, Baltimore, MD, United States; ^4^Institute of Preventive Medicine and Public Health, Hanoi Medical University, Hanoi, Vietnam; ^5^Institute for Global Health Innovations, Duy Tan University, Da Nang, Vietnam; ^6^Faculty of Medicine, Duy Tan University, Da Nang, Vietnam; ^7^Center for Digital Health, Medical Science Research Institute, Kyung Hee University College of Medicine, Seoul, Republic of Korea; ^8^Department of Pediatrics, Kyung Hee University Medical Center, Kyung Hee University College of Medicine, Seoul, Republic of Korea; ^9^TIMC-IMAG, UMR 5525 Joint Research Unit, Centre National de Recherche Scientifique, National Center for Scientific Research, Université Grenoble-Alpes, Grenoble, France

**Keywords:** patient safety, clinical errors, health services research, fear, healthcare workers, burnout syndrome, mental health, cross-sectional study

## Abstract

**Background:**

The fear of clinical errors among healthcare workers (HCW) is an understudied aspect of patient safety. This study aims to describe this phenomenon among HCW and identify associated socio-demographic, professional, burnout and mental health factors.

**Methods:**

We conducted a nationwide, online, cross-sectional study targeting HCW in France from May to June 2021. Recruitment was through social networks, professional networks, and email invitations. To assess the fear of making clinical errors, HCW were asked: “During your daily activities, how often are you afraid of making a professional error that could jeopardize patient safety?” Responses were collected on a 7-point Likert-type scale. HCW were categorized into “High Fear” for those who reported experiencing fear frequently (“once a week,” “a few times a week,” or “every day”), vs. “Low Fear” for less often. We used multivariate logistic regression to analyze associations between fear of clinical errors and various factors, including sociodemographic, professional, burnout, and mental health. Structural equation modeling was used to explore how this fear fits into a comprehensive theoretical framework.

**Results:**

We recruited a total of 10,325 HCW, of whom 25.9% reported “High Fear” (95% CI: 25.0–26.7%). Multivariate analysis revealed higher odds of “High Fear” among males, younger individuals, and those with less professional experience. High fear was more notable among physicians and nurses, and those working in critical care and surgery, on night shifts or with irregular schedules. Significant associations were found between “High Fear” and burnout, low professional support, major depressive disorder, and sleep disorders.

**Conclusions:**

Fear of clinical errors is associated with factors that also influence patient safety, highlighting the importance of this experience. Incorporating this dimension into patient safety culture assessment could provide valuable insights and could inform ways to proactively enhance patient safety.

## Introduction

Clinical errors are an ongoing challenge to health care workers (HCW) and health care institutions worldwide (1). These errors arise from the complex interplay of latent and active failures, and pose a significant threat to patient safety and healthcare quality (2). They contribute to patient harm, which has been cited as the 14th leading cause of the global disease burden (3), and in the United States, the eighth leading cause of patient mortality (4, 5). Beyond the harm these errors cause to patients, errors also profoundly affect HCW, who often endure emotional and psychological distress as “second victims” (6). Despite ongoing efforts to minimize harmful errors, their occurrence is a persistent concern (7). New approaches are needed address them so as to enhance patient safety in healthcare.

In response to these challenges, this paper explores a new indicator that may help to enhance patient safety—the fear among HCW of committing an error that harms a patient. Unlike previous approaches that primarily focus on the identification and documentation of errors, this approach emphasizes gaining an understanding the internal experiences and perceptions of HCW. The fear of error has been overlooked in patient safety culture assessments, and could provide valuable insights into both working conditions and the psychological state of HCW (8, 9). Fear of clinical error could serve as an indicator of the pressures and challenges HCW face, which also pose a threat to patient safety. Indeed, clinicians who feel guilty after a medical error often experience parallel feelings of fear—fear for their reputation, their job, their license, and their future as well as that of their patient (10). Fear is associated with other adverse emotions, including guilt, shame, anxiety, and depression, and these are common among physicians and other healthcare providers following a medical error (11). Moreover, studies have reported that the fear associated with errors is not just the fear of legal action in medical decision-making, but rather the fear of causing harm (12). Furthermore, recognizing and addressing this experience among HCW may be crucial in the context of increasing concerns about HCW burnout and mental health (13). Fear of medical errors has been reported as a significant risk factor for burnout (14).

This study aims to describe the fear of clinical error among HCW, to identify associated socio-demographic, professional, burnout and mental health factors, and to explore the interrelationship among these factors using a Structural Equation Modeling (SEM).

## Methods

### Study design and participants

This was a cross-sectional survey-based study of HCW based on the AMADEUS study in France (15). AMADEUS (“AMéliorer l'ADaptation à l'Emploi pour limiter la soUffrance des Soignants” or “Improve Employment Adaptation to Limit Healthcare Workers' Suffering”) was a nationwide, online, cross-sectional survey conducted in public and private healthcare facilities across France. The survey period spanned from May 2nd to June 30th, 2021. Recruitment of participants was achieved through outreach via social networks, professional networks, and email invitations. The detailed protocol has been published (15). The primary goal was to determine the prevalence of burnout among HCW. Secondary objectives included examining the relationship between burnout, various professional and psychosocial factors (including fear of clinical errors), and mental health (16–18). The study adheres to ethical principles for medical research involving human subjects, in compliance with the French Jardé law (19), and was approved by an independent ethical committee (IRB No. C08/21.01.06.93911). In line with the Safety Culture Theory (20), which posits that an organization's culture significantly influences its members' attitudes and behaviors related to safety, our study sought to explore how the fear of clinical errors among HCW reflects the broader safety culture within healthcare settings in France.

### Evaluation criteria and collected variables

To assess the fear of making clinical errors, the survey asked: “During your daily activities, how often are you afraid of making a professional error that could jeopardize patient safety?” Responses were gathered on a 7-point Likert scale. Participants were categorized into two groups: those indicating frequent fear (“once a week,” “a few times a week,” or “every day”) were placed in the “High Fear” group, while others were included in the “Low Fear” group (“never,” “at least a few times a year,” “at least once a month,” or “a few times a month”).

Socio-demographic variables included age (in years), sex (male, female), and personal social support (presence of a partner: yes, no). Professional data encompassed the type of profession (physicians, nurses, nurse assistants, health executives, and other professions), and the length of time HCW had been in their profession. Departmental data spanned sectors including surgery, medical, critical care, and other departments. Job characteristics, included whether participants had a full-time job, a night shift job, worked night shifts, and consistent schedules. Burnout was assessed using the French version of the 22-item Maslach Burnout Inventory (MBI) scale, which evaluates three key dimensions: emotional exhaustion, depersonalization, and low personal accomplishment (21, 22). Burnout was operationalized as a binary variable. A participant was categorized as experiencing burnout if they met or exceeded the cut-off scores in at least one of the three dimensions, defined as ≥30 for emotional exhaustion, ≥12 for depersonalization, and ≤ 40 for personal accomplishment (21). Low professional support was measured using the Karasek isostrain measure, with the threshold based on the combination of job strain and a social support score of <24 (23). The assessment of mental health factors was based on the presence of major depressive disorders using the Center for Epidemiologic Studies- Depression Scale (CES-D) (24) [probable depression is defined by a score ≥17 in men and ≥23 in women (25)], sleep disorders (Pittsburgh Sleep Quality Index questionnaire score>5) (26, 27), tobacco smoking (self-reported assessment: yes, no), and hazardous drinking (CAGE questionnaire score≥2) (28).

### Statistical analysis

All variables were represented by mean values and standard deviation (SD) for continuous data, and by frequency distributions for categorical data. To compare HCW categorized into “High Fear” and “Low Fear” groups, chi-square tests were applied for categorical variables. For continuous variables, Student's *t*-tests or Mann-Whitney tests were utilized according to their distribution. To explore the factors associated with “High Fear,” a multivariate logistic regression model was employed, presenting adjusted Odds Ratios (aORs) and 95% confidence intervals (95% CIs). Variables demonstrating significant associations in the univariate analysis were incorporated into the multivariate model. A significance of two-sided *p* < 0.05 was used. The data was analyzed with the SPSS (version 20.0; IBM, USA).

**Figure 1:**
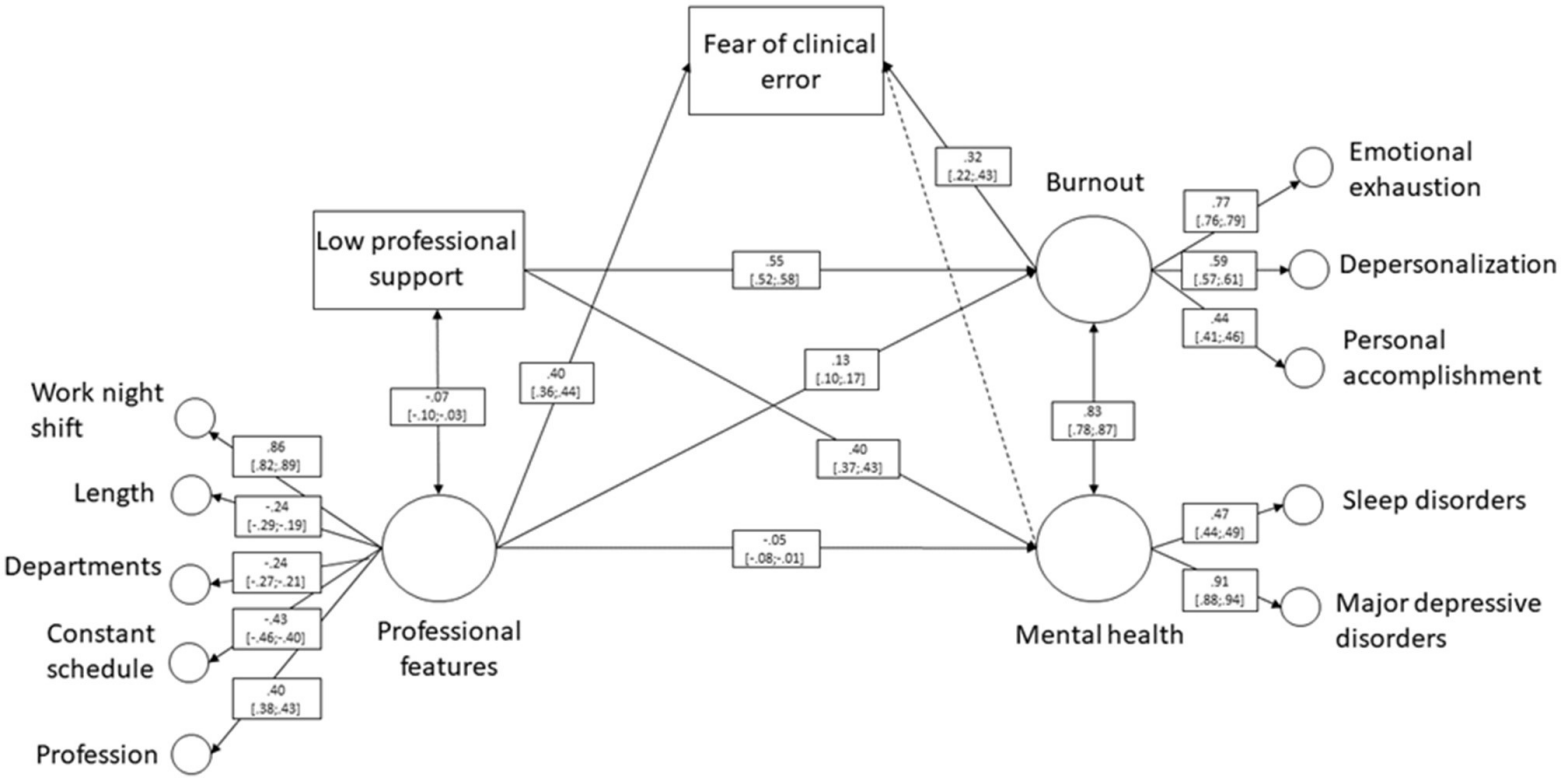
Structural equation modeling diagram.

We employed a SEM approach to examine how this fear fits into a comprehensive theoretical framework. The SEM was conducted to identify the direct, indirect, and overall effects of factors associated with the fear of clinical error (Figure 1). We hypothesized that professional features and professional support would directly impact the risk of burnout and mental health (16, 17). In addition, we expected professional features, mental health and burnout to be related to the fear of clinical error; and burnout to mediate the relationship between professional features and the fear of clinical error. Burnout and mental health disorders would be interrelated bidirectionally (15), as would professional features and professional support. Standardized path coefficients (β) and 95% CIs were reported. The weighted least squares means and variance adjusted (WLSMV) robust estimator was employed, as recommended for modeling latent factors with both categorical (binary and ordinal) and continuous variables, even in the absence of normal distribution. The Comparative Fit Index (CFI), the Tucker–Lewis Index (TLI), the Root Mean Square Error of Approximation (RMSEA), and the Standardized Root Mean Square Residual (SRMR) were used to assess the overall model fit. A CFI and TLI ≥ 0.90, an RMSEA ≤ 0.08, and an SRMR ≤ 0.08 indicate a good model fit (29). In addition to the statistical significance of the path coefficients, the strength of the relationships was considered, classifying them as weak (<0.2), moderate (0.2–0.5), or strong (>0.5) (30). This analysis was performed using R software version 4.1.3, with the Lavaan package (31).

## Results

A total of 10,325 HCW participated in the study, comprising 1,969 physicians (19.1%), 1,768 health executives (17.1%), 2,819 nurses (27.3%), 847 nurse assistants (8.2%), and 2,922 other health professionals (28.3%). The total size of the population HCW from which this sample was drawn (denominator) is unknown. The average age was 42.3 years (SD = 10.8), with 1,989 male (19.3%; Table 1).

**Table 1:** Descriptive data of respondents, France, 2021 (*N* = 10,325).

	***N* or mean**	**(% or SD)**	**% of “high clinical error fear”**
**Socio-demographic data**			
Age (years)	42.3	(10.8)	-
**Sex**			
Male	1,989	(19.3%)	31.3%
Female	8,336	(80.7%)	24.6%
**Personal social support**			
Partner	7,692	(74.5%)	25.2%
No partner	2,633	(25.5%)	28.0%
**Professional data**			
**Profession**			
Physician	1,969	(19.1%)	39.9%
Nurse	2,819	(27.3%)	31.6%
Nurse assistant	847	(8.2%)	19.6%
Health executive	1,768	(17.1%)	11.9%
Other professions	2,922	(28.3%)	21.2%
Length of time in the profession (years)	13.9	(10.1)	-
**Length of time in the profession**			
≤ 1 year	656	(6.4%)	36.9%
>1 year	9,669	(93.6%)	25.1%
**Departments**			
Surgery departments	1,067	(10.3%)	30.6%
Medical departments	4,924	(47.7%)	27.2%
Critical care departments	844	(8.2%)	37.6%
Other departments	3,490	(33.8%)	19.8%
**Job characteristics**			
Full-time job	8,630	(83.6%)	26.0%
No full-time job	1,695	(16.4%)	25.1%
Night shift job	648	(6.3%)	31.0%
No night shift job	9,677	(93.7%)	25.5%
Work night shift	3,046	(29.8%)	38.9%
No work night shift	7,161	(69.4%)	20.4%
Constant schedule	5,435	(52.6%)	20.2%
No constant schedule	4,890	(47.4%)	32.2%
Burnout	5,712	(55.3%)	32.4%
No Burnout	4,613	(44.7%)	17.9%
Emotional exhaustion	3,004	(29.1%)	39.2%
No emotional exhaustion	7,321	(70.9%)	20.4%
Depersonalization	2,353	(22.8%)	42.5%
No depersonalization	7,972	(77.2%)	21.0%
Low personal accomplishment	3,564	(34.5%)	30.1%
Personal accomplishment	6,761	(65.5%)	23.7%
Low professional support	3,220	(31.2%)	30.6%
Professional support	7,105	(68.8%)	23.7%
Major depressive disorders	3,122	(30.2%)	36.6%
No major depressive disorders	7,203	(69.8%)	21.3%
Sleep disorders	3,829	(37.2%)	29.9%
No sleep disorders	6,486	(62.8%)	23.5%
Tobacco smoking	2,115	(20.5%)	25.1%
No tobacco smoking	8,210	(79.5%)	26.1%
Hazardous drinking	1,925	(18.6%)	31.3%
No hazardous drinking	8,400	(81.4%)	24.6%

**Figure 2:**
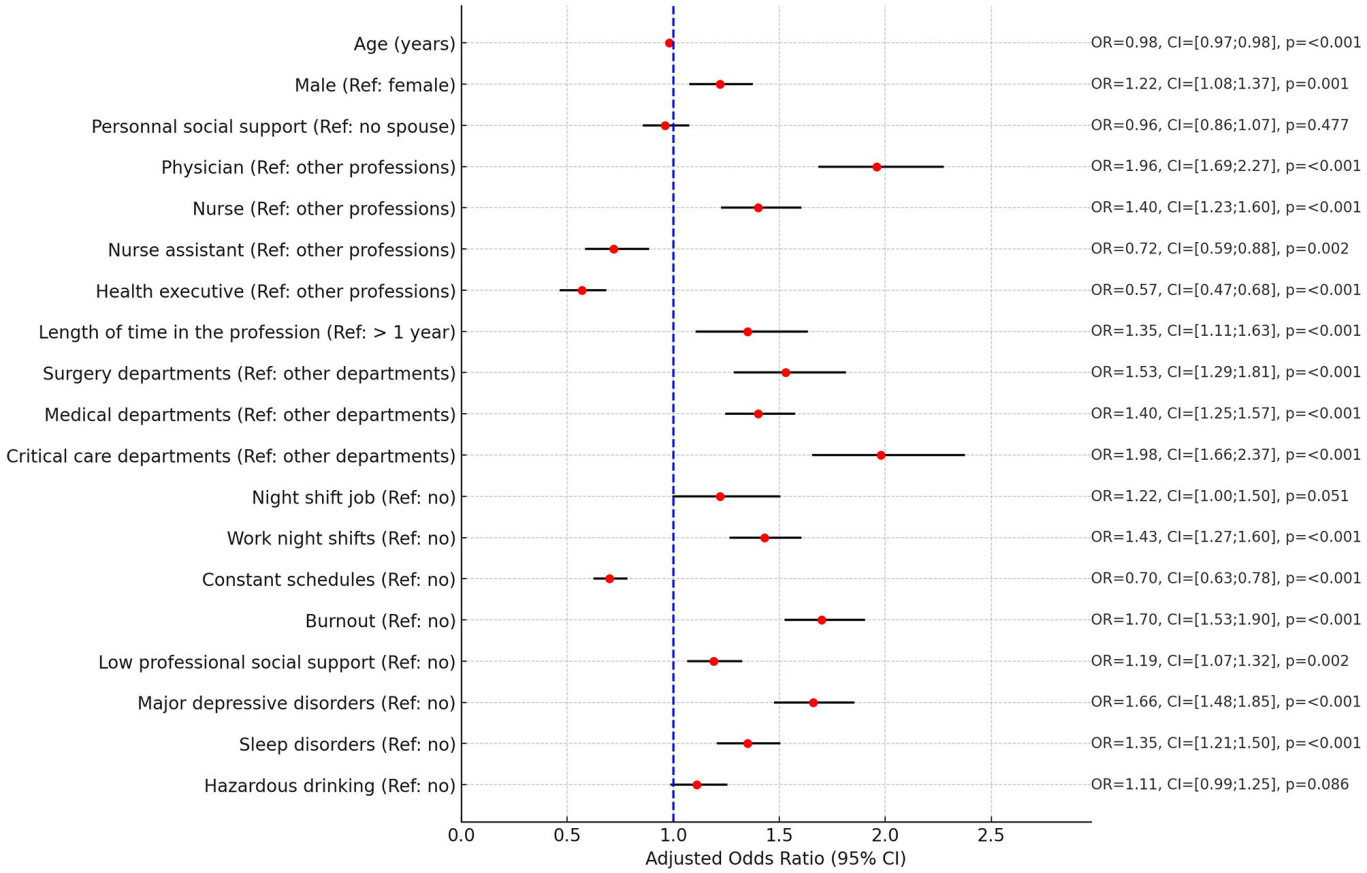
Forest plot of multivariate analysis on factors associated with fear of clinical errors. OR, Adjusted Odds Ratio; 95% CI, 95% confidence intervals.

Overall, 25.9% of HCW (95% CI: 25.0–26.7%) reported “High Fear.” The factors associated with fear of clinical error are shown in Table 2 (univariate analysis) and Figure 2 (multivariate analysis). Multivariate analysis revealed that “High Fear” was more prevalent among male HCW, younger individuals, and those with less professional experience. Heightened fear was notable among physicians and nurses, as well as those working in critical care, surgery, and medical departments. HCW working night shifts or having irregular schedules also showed higher levels of fear. Additionally, significant associations were found between “High Fear” and the presence of burnout, low professional support, major depressive disorders, and sleep disorders.

**Table 2:** Factors associated with clinical error fear: univariate analysis.

	**“Low fear” group**		**“High fear” group**		
	**(*****N*** = **7,652)**		**(*****N*** = **2,673)**		
	***N* or mean**	**(% or SD)**	***N* or mean**	**(% or SD)**	***P*-value**
**Socio-demographic data**					
Age (years)	43.3	(10.7)	39.4	(10.8)	<0.001
Sex					<0.001
Male	1,366	(17.9%)	623	(23.3%)	
Female	6,286	(82.1%)	2,050	(76.7%)	
Personal social support (partner)	5,757	(75.2%)	1,935	(72.4%)	0.004
**Professional data**					
Profession					<0.001
Physician	1,184	(15.5%)	785	(29.4%)	
Nurse	1,927	(25.2%)	892	(33.4%)	
Nurse assistant	681	(8.9%)	166	(6.2%)	
Health executive	1,558	(20.4%)	210	(7.9%)	
Other professions	2,302	(30.1%)	620	(23.2%)	
Length of time in the profession (years)	14.6	(10.2)	12.0	(9.7)	<0.001
Length of time in the profession ( ≤ 1 year)	414	(5.4%)	242	(9.1%)	<0.001
Departments					<0.001
Surgery departments	740	(9.7%)	327	(12.2%)	
Medical departments	3,586	(46.9%)	1,338	(50.1%)	
Critical care departments	527	(6.9%)	317	(11.9%)	
Other departments	2,799	(36.6%)	691	(25.9%)	
**Job characteristics**					
Full-time job	6,382	(83.4%)	2,248	(84.1%)	0.402
Night shift job	447	(5.8%)	201	(7.5%)	0.002
Work night shifts	1,862	(24.6%)	1,184	(44.8%)	<0.001
Consistent schedules	4,436	(56.7%)	1,099	(41.1%)	<0.001
Burnout	3,863	(50.5%)	1,849	(69.2%)	<0.001
Emotional exhaustion	1,826	(23.9%)	1,178	(44.1%)	<0.001
Depersonalization	1,353	(17.7%)	1,000	(37.4%)	<0.001
Low personal accomplishment	2,492	(32.6%)	1,072	(40.1%)	<0.001
Low professional support	2,234	(29.2)	986	(36.9%)	<0.001
**Mental health data**					
Major depressive disorders	1,980	(25.9%)	1,142	(42.7%)	<0.001
Sleep disorders	2,690	(35.2%)	1,149	(43.0%)	<0.001
Tobacco smoking	1,584	(20.7%)	531	(19.9%)	0.357
Hazardous drinking	1,322	(17.3%)	603	(22.6%)	<0.001

The theoretical model provided adequate fit [$\chi_{\left(47\right)}^{2}$ = 1,087.10, *p*-value <0.001, CFI = 0.934, TLI = 0.908, RMSEA = 0.047% CI [0.044–0.049], and SRMR = 0.059; Table 3]. Overall, nearly all the paths in the theoretical model were significant. As expected, professional features and professional support had significant direct effects on the risk of burnout and mental health. Burnout and mental health were interrelated bidirectionally, as well as professional features and professional support. Both professional features and burnout had significant direct effects on the fear of clinical error, but the direct effect of mental health was not significant. In addition, we also found the mediation effect of burnout on the relationship between professional features and the fear of clinical error.

**Table 3:** Total, direct and indirect effects of model paths.

**Model paths**	**Total effects**	**Direct effects**	**Indirect effects**
	**95% CI**	**95% CI**	**95% CI**
Mental health -> Fear of clinical error	0.03	0.03	-
	[−0.07; 0.14]	[−0.07; 0.14]	
Professional features -> Fear of clinical error	0.45	0.40	0.04
	[0.41; 0.48]	[0.36; 0.44]	[0.03; 0.06]
Burnout -> Fear of clinical error	0.32	0.32	-
	[0.22; 0.43]	[0.22; 0.43]	
Professional features -> Burnout	0.13	0.13	-
	[0.10; 0.17]	[0.10; 0.17]	
Professional support -> Burnout	0.55	0.55	-
	[0.52; 0.58]	[0.52; 0.58]	
Professional features -> Mental health	−0.05	−0.05	-
	[−0.08; −0.01]	[−0.08; −0.01]	
Professional support -> Mental health	0.40	0.40	-
	[0.37; 0.43]	[0.37; 0.43]	

## Discussion

Addressing and reducing preventable patient harm remains a significant global public health challenge (1). In this national survey of French HCW, a quarter reported “High Fear,” reflecting a high prevalence and pervasive atmosphere of perceived risk. This investigation of HCW's experience of the risk of error reveals interrelated factors, including socio-professional characteristics, lack of professional support, burnout, and mental health. This supports the idea that focusing on the fear of clinical errors among HCW can complement and enhance existing strategies for mitigating preventable patient harm (32, 33).

Fear of clinical error was associated with factors demonstrated to influence patient safety, underscoring the relevance of HCW experiential perspective in safety considerations. Younger age and a short tenure in the profession can be attributed to a lack of experience and a need for enhanced guidance. Previous studies have indicated that many young professionals feel unprepared for their roles, which reflects and elevated risk to patient safety due to the potential for individual errors (34). This supports the imperative to improve job adaptation, qualification training, and provide structured professional mentorship (34). To our knowledge, the current literature has not provided evidence on studies differences between men and women on the fear of clinical errors. Future research will be necessary to explore and understand the explanatory factors behind this finding. If this is confirmed, it will be important to consider this in designing support programs. Physicians and nurses experience higher levels of fear regarding clinical errors compared to other healthcare professionals. This can be attributed to their critical roles in patient care and decision-making, which carry greater potential for involvement in harmful errors. Their positions entail a heightened sense of accountability as well as accompanying pressure of potential repercussions such as punishment, disciplinary actions, and job loss. These may all contribute to an increased fear of making clinical errors (35). As might be expected, dritical care and surgery departments were also associated with high levels of fear of clinical errors. This is likely attributable to the complex and life-critical nature of the tasks involved in these settings (36, 37). The demanding environment, characterized by urgent decision-making and the potential for severe adverse outcomes, amplifies this fear among HCW. This underscores the need for robust support systems and a strong safety culture in these high-stakes areas (38). Working night shifts or maintaining irregular schedules is associated with fatigue (39), which may account for the heightened fear of clinical errors. This study also showed that sleep disorders and major depressive disorders were not directly associated with the fear of clinical error. Burnout, major depressive disorders and sleep disorders are interdependent (16, 18, 40–42) and thus collectively influence the fear of clinical error. Burnout is associated with and can contribute to a range of mental disorders, such as sleep problems, depression, anxiety. Similarly, pre-existing mental disorders can increase the risk of burnout. Previously identified risk factors for burnout include high workloads, lack of autonomy, poor professional support and low rewards (15). The role of professional support in preventing and mitigating burnout is crucial, and can help reduce the risk of depressive disorders and sleep disorders among HCW (15, 43). To prevent burnout, work organization interventions and both individual and collective support measures are needed. This finding underscores the potential utility of peer-based interventions. This is in line with findings from previous studies highlighting the effectiveness of peer-support programs in providing psychological first aid and emotional support to HCW like RISE (Resilience In Stressful Events) (44–46).

Identifying the root causes of the fear of making errors could help healthcare organizations proactively mitigate potential problems before they are realized as actual errors. This could complement traditional reactive strategies, which usually concentrate on addressing errors after they have occurred. Notably, fear of consequences is the most reported barrier for reporting errors (47). Consequently, by focusing on fear rather than solely on errors could help health care organizations to foster a more transparent reporting culture. This encourages HCW to report potential risks and errors, thereby facilitating learning from incidents. By fostering such an approach, healthcare organizations create an environment where not only are immediate safety concerns addressed, but also nurture a broader culture of patient safety. In this culture, continuous learning and improvement are integral to healthcare practice.

The limitations of this study are similar to those affecting other online and cross-sectional surveys. With a cross-sectional design and despite the use of SEM, no causal relationship can be drawn. The measurement of fear of clinical error was based on an *ad-hoc* question. To our knowledge, there is no validated measure for this specific concept. However, the absence of missing data for this question provides support for its acceptability. Future studies should investigate the relationship between this indicator and measures related to patient safety culture and adverse events. We cannot calculate a response rate and we cannot exclude the potential for sampling bias. Participants off work for depression, burnout or other causes may not have received professional mailings. However, we have disseminated this survey at multiple timepoints in attempts reach these participants. Additionally, the survey was disseminated through social networks and our geographical coverage seems adequate to increase geographical exhaustivity. The threshold for categorizing 'High' and 'Low' fear groups is somewhat arbitrary and may underrepresent the issue. This categorization simplifies the complex spectrum of fear experiences. Notably, experiencing fear “at least once a month” or “a few times a month” is also concerning. The three sociodemographic variables—age, sex, and personal social support—could not be effectively synthesized into a latent variable. Consequently, they could not be included in the SEM and were not examined as such in the multivariable analysis. A last shortcoming of our study is the absence of data on whether the subjects committed clinical errors, how those errors were handled, and the time elapsed from the error to the study. Future research needs to incorporate a qualitative methodology, such as focus groups or simulation scenarios, to address this gap. This approach would provide deeper insights into the fear of making mistakes, as currently, there is no specific questionnaire addressing this construct.

## Conclusion

The fear of clinical error among HCW is associated with key factors influencing patient safety, highlighting the importance of this aspect of HCW experience in safety considerations. Incorporating this concepts into patient safety culture assessment could add valuable insights and may serve as a means to proactively enhance patient safety in healthcare settings.

## Data availability statement

The raw data supporting the conclusions of this article will be made available by the authors, without undue reservation.

## Ethics statement

The studies involving humans were approved by IRB Comité de protection des personnes No. C08/21.01.06.93911. The studies were conducted in accordance with the local legislation and institutional requirements. The participants provided their written informed consent to participate in this study.

## Author contributions

LB: Conceptualization, Supervision, Validation, Writing – original draft, Writing – review & editing. AW: Validation, Writing – review & editing. SF: Formal analysis, Methodology, Writing – review & editing. BT: Validation, Writing – review & editing. YB: Validation, Writing – review & editing. TN: Validation, Writing – review & editing. DY: Validation, Writing – review & editing. PA: Validation, Writing – review & editing. GL: Conceptualization, Data curation, Supervision, Validation, Writing – review & editing. BB: Validation, Writing – review & editing. GF: Methodology, Project administration, Supervision, Validation, Writing – original draft, Writing – review & editing.
